# The phage anti-restriction induced system: new insights into bacterial immunity and bacteriophage escape strategies

**DOI:** 10.1038/s41392-024-01995-x

**Published:** 2024-10-05

**Authors:** Yi Zhong, Volker M. Lauschke

**Affiliations:** 1 Department of Physiology and Pharmacology and Center of Molecular Medicine, Karolinska Institutet and Karolinska University Hospital, Stockholm, Sweden; 2 Pharmaceutical Informatics Institute, College of Pharmaceutical Sciences, Zhejiang University, Hangzhou, Zhejiang China; 3 Dr Margarete Fischer-Bosch Institute of Clinical Pharmacology, Stuttgart, Germany; 4 Department of Pharmacy, the Second Xiangya Hospital, Central South University, Changsha, China

**Keywords:** Microbiology, Immunology

Two recent publications in *Nature* reveal the detailed structural mechanisms underlying the recognition of infection by the bacterial Phage Anti-Restriction Induced System (PARIS).^1,2^ These findings are important as they show a new strategy for the recognition of viral infections, shed light on principles of host-virus co-evolution and might pave the way for the development of new antimicrobial strategies.

PARIS belongs to the toxin-antitoxin (TA) systems, which are small genetic elements that are involved in a plethora of bacterial functions, including plasmid maintenance, biofilm production, persistence, virulence and defense against bacteriophages. They are common in nearly all prokaryotic phyla and >200,000 putative TA modules have been identified today across 41,610 analyzed genomic assemblies.^3^ Among the different classes, type II TA systems, in which an antitoxin protein binds and inhibits the toxin protein, are best characterized. The PARIS system constitutes a bacterial defense mechanism consisting of an ABC ATPase protein (AriA) and a TOPRIM/OLD-family nuclease (AriB). However, the molecular mechanisms of how PARIS inhibits bacteriophage propagation and how phages can escape PARIS immunity remained unclear.

Using cryo-EM structures with resolution down to 3.1 Å, the authors show that the antitoxin AriA forms a stable homohexameric ring structure that can tightly bind and sequester up to three units of the AriB toxin, effectively inhibiting its nuclease activity. Upon infection, bacteria use restriction-modification (RM) and CRISPR/Cas systems to cleave foreign DNA thereby blocking phage propagation. To escape this “first-line” defense mechanism, bacteriophages can express factors that counter host defenses. In the case of T7, the phage-encoded protein Ocr (‘overcome classical restriction’) can bind the restriction complex and inhibit its recognition of phage DNA. Notably, PARIS not only recognizes T7-encoded Ocr but also T4-encoded structurally dissimilar anti-restriction protein Arn25. The ability to identify such diverse viral inhibitors of restriction defense mechanisms is likely due to electrostatic interactions between positively charged residues at AriA and negatively charged surfaces on Ocr and Arn25.

The new cryo-EM data indicate that upon recognition of Ocr, AriA undergoes a conformational change that decreases its affinity for AriB, which results in its release from the AriA-AriB complex. Subsequently, AriB dimerizes into homodimers, which significantly enhances its nuclear activity. The authors demonstrate that, once activated, AriB triggers abortive infection by blocking bacterial translation. Specifically, AriB cleaves bacterial lysine tRNA (tRNA^Lys^), thereby blocking both bacterial growth and phage replication. This structural information thereby provides the molecular basis for the rapid and effective bacterial response against phage infections.

Strikingly, T5 bacteriophages could escape PARIS immunity and using a series of elegant experiments, the authors could show that phage-encoded tRNA^Lys^, which harbors substitution mutations in the anticodon stem loop, was not targeted by AriB.^2^ These viral tRNA^Lys^ can thus compensate for the depletion of bacterial tRNA^Lys^, consistent with previous studies suggesting that phage-encoded tRNAs have evolved to be insensitive to host anticodon nucleases.^4^

**Fig. 1:**
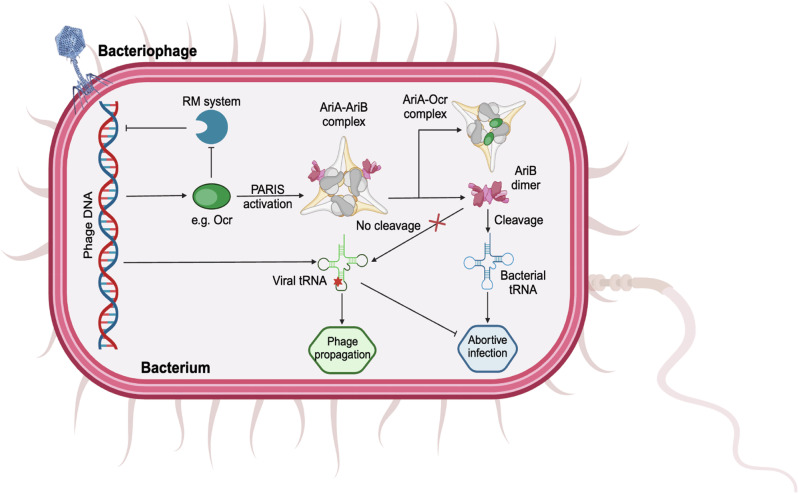
Schematic of activation and effector functions of the PARIS defense system. Negatively charged phage-encoded proteins, such as Ocr, activate PARIS immunity by displacing AriB from the AriA-AriB complex. Unbound AriB can then cleave bacterial tRNAs to terminate translation, resulting in growth arrest and abortive infection. Phage-encoded tRNAs with mutations near the stem loop (indicated by red asterisk) are resistant to AriB-mediated degradation and can overcome this defense mechanism, resulting in phage escape. RM restriction-modification system. Created in BioRender (BioRender.com/r10w201)

The new crystal structures of the PARIS TA system and the functional revelations they provide give further molecular insights into of the bacteria-bacteriophage arms race. Specifically, the structural analysis of AriA and AriB offers a molecular framework for understanding how bacteria utilize these proteins to mount a rapid and effective defense against phage infections. The available data is compatible with a model in which infection is commonly recognized by RM-based defense mechanisms (Fig. 1). To overcome this defense, bacteriophages can express inhibitors of restriction, such as Ocr, which act as DNA decoys and block phage DNA recognition. Activation of PARIS by phage RM countermeasures induces bacterial cell death, thus preventing the spread of phage infection to nearby cells. In turn, phages have evolved strategies, such as the encoding of viral degradation-resistant tRNAs that can facilitate phage propagation even if PARIS is triggered and degrades bacterial tRNAs to block translation. As such, the findings not only add a new dimension to our understanding of bacterial immunity but also highlight the evolutionary pressures that drive the diversification of both bacterial defense mechanisms and phage countermeasures.

The new results significantly advance our understanding of bacterial immune mechanisms against bacteriophages and raise appealing questions for future research. Specifically, they indicate that contrary to previous hypotheses,^5^ viral tRNAs do not (only) seem to increase phage fitness by optimizing the available tRNA pool to the codon usage in the viral genome, but they also present a strategy to escape bacterial immunity. In the future, it will be interesting to comprehensively profile the proteins that can trigger PARIS across different phages. The fact that both currently identified PARIS activators are inhibitors of bacterial RM defense systems, furthermore, raises the question whether also functionally distinct viral proteins with similar biophysical properties to Ocr and Arn25 might trigger PARIS. These findings might provide insights into the evolutionary origins—did PARIS co-evolve as a second line of defense specifically against phages that had acquired countermeasures against bacterial RM systems or does PARIS constitute a parallel defense mechanism that developed in parallel to other bacterial defense mechanisms? Additionally, it will be exciting to investigate the co-evolution of tRNA degradation strategies in host immunity and the tRNA pools encoded in viral genomes. Taken together, these findings contribute to the broader knowledge of microbial interactions and have potential implications for the development of new antimicrobial strategies, for instance by purposeful activation of TA systems of pathogenic bacteria via tailored infection mimetics.
